# ‘The public health turn on violence against women’: analysing Swedish healthcare law, public health and gender-equality policies

**DOI:** 10.1186/s12889-020-08766-7

**Published:** 2020-05-24

**Authors:** Ann Öhman, Monica Burman, Maria Carbin, Kerstin Edin

**Affiliations:** 1 Umeå Centre for Gender Studies (UCGS), Umeå University, SE-901 87 Umeå, Sweden; 2 Department of Epidemiology and Global Health, Umeå University, Umeå, Sweden; 3 Police Education Unit at Umeå University, Umeå, Sweden; 4 Department of Nursing, Umeå University, Umeå, Sweden

**Keywords:** Violence against women, Intimate partner violence, Public health, Gender equality, Policy, Healthcare services, Sweden, Governing, Healthcare law

## Abstract

This article focuses on policy and law concerning violence against women as a public health issue. In Sweden, violence against women is recently recognized as a public health problem; we label this shift “The public health turn on violence against women”. The new framing implies increased demands on the Swedish healthcare sector and its’ ability to recognise violence and deal with it in terms of prevention and interventions. The aim was to describe and discuss the main content and characteristics of Swedish healthcare law, and national public health and gender-equality policies representing the public health turn on violence against women. Through discursive policy analysis, we investigate how the violence is described, what is regarded to be the problem and what solutions and interventions that are suggested in order to solve the problem. Healthcare law articulates violence against women as an ordinary healthcare issue and the problem as shortcomings to provide good healthcare for victims, but without specifying what the problem or the legal obligation for the sector is. The public health problem is rather loosely defined, and suggested interventions are scarce and somewhat vague. The main recommendations for healthcare are to routinely ask patients about violence exposure. Violence against women is usually labelled “violence within close relationships” in the policies, and it is not necessarily described as a gender equality problem. While violence against women in some policy documents is clearly framed as a public health problem, such a framing is absent in others, or is transformed into a gender-neutral problem of violence within close relationships. It is not clearly articulated what the framing should lead to in terms of the healthcare sector’s obligations, interventions and health promotions, apart from an ambivalent discourse on daring to ask about violence.

## Background

This article focuses on policy and law concerning men’s violence against women as a public health issue. Women exposed to violence have long sought help for different health problems related to the violence, such as headache and long-term pain. They have however had difficulties in receiving adequate help, as the issue of violence has not been on the policy agenda in the healthcare system and health professionals have had few guidelines and recommendations to adhere to. Although research and international bodies have described violence against women as a severe public health problem at least since the 1990s [1], it is only recently that it has become more widely framed and recognized as a public health problem in Sweden. We have noticed this new framing of violence against women in several official policy documents and we label it “The public health turn on violence against women”. Previously, violence against women was understood primarily as a social and legal problem, or even as a private problem, rather than framed as a health matter [2]. The shift towards public health is our point of departure for this article.

We can clearly see the shift in several reports on violence against women, reports emanating from authorities other than the health sector. For instance, in a study from The National Centre for Knowledge on Men’s Violence against Women (NCK) it is emphasized that violence against women is a public health matter with severe consequences for victims of violence in terms of physical and mental health problems [3]. In a government report, it is stated that violence ‘within close relationships’ is a public health matter and it concludes that coordinated actions between several societal institutions are needed, among them the healthcare sector [4]. From a legal perspective, the shift is represented by the fact that a need to nationally govern the healthcare sector regarding violence for the first time was identified and resulted in binding national regulations in 2014 [5]. The reframing towards public health implies increased demands on the health system and health services and their ability to face violence against women as a health problem and deal with it in terms of prevention and interventions.

To our knowledge, no studies on policy and law have as yet focused on the public health turn on violence against women in Sweden. We aim therefore to fill this knowledge gap by describing and problematizing the main content and characteristics of Swedish healthcare law, public health and gender-equality policies representing the public health turn on violence against women.

### Violence against women as a public health issue

Violence against women is internationally described as a global public health problem with severe consequences, not only for the woman herself, but also for her children. Besides individual suffering, violence against women also contributes to high societal costs in terms of legal procedures, healthcare treatment and social problems [6]. Violence against women was recognized as a global public health issue when in 1996 the World Health Assembly adopted a resolution which declared that violence is a leading public health problem worldwide. They also highlighted the urgent need to address violence against women and girls by using a gender perspective when analysing its causes and magnitudes towards the goal of elimination [1, 2]. Worldwide, it is estimated that about one in three women, after the age of 15, experience physical and/or sexual violence from an intimate partner during their life-course [6]. The reported prevalence of partner violence varies between countries and is correlated with gender inequalities, which exercise an influence on norms, legislation, everyday life and access to resources, resulting in substantially more disadvantages for women than for men [7]. Another explanation for the widely different prevalence figures is methodological variations in research design, as well as varying safety and ethical arrangements that might influence women’s willingness to disclose exposure to violence [8]. The WHO highlights the importance of the healthcare sector expanding its role in regard to violence protection. García-Moreno and colleagues [9, 10] also emphasize governments’ responsibility to develop action plans, including education and other fundamental actions against gendered structures that sustain inequality between men and women, in order to prevent and counteract violence against women and girls.

In Sweden, almost every second woman has reported life-time exposure to some kind of serious violence, of which 65% was sexual harassment and 20% was sexual violence. Fourteen percent of the women reported violence by a former or present intimate partner [3]. A later publication from the same NCK study revealed that 6% of women reported exposure to at least one kind of violence in the previous year [11]. The Swedish government has commissioned the National Board of Health and Welfare (the Board) to support and develop the undertaking by social services and healthcare regarding men’s violence against women during the period 2017–2026 [12]. The policy analysis that we conduct in this paper is therefore important for highlighting how violence is defined and articulated in the policy documents.

### An international legal obligation

Violence against women is a violation of women’s human rights. The human rights law obligations for the state of Sweden to act have sharpened substantially during the last few years. Sweden has ratified the legally binding 2011 Council of Europe Convention on preventing and combating violence against women and domestic violence (the Istanbul convention) [13]. Moreover, in 2017 the CEDAW committee, the United Nations body monitoring the Convention on the Elimination of All Forms of Discrimination against Women, claimed that a binding international legal norm on state responsibility has evolved [14]. Both the CEDAW jurisprudence and the Istanbul Convention address the healthcare sector as an important societal actor and stress the importance of access to healthcare, adequately resourced services and trained professionals [15, 16]. Swedish healthcare law has only recently begun to address men’s violence against women, and, as will be illustrated below, only in a minor and rather cautions manner.

### The central position of gender-equality

Gender equality has a central position in Swedish politics. One could say that the discourse of gender equality, at least temporarily, has achieved a hegemonic position, meaning that almost no one argues against it [17]. The strong discourse of gender equality and gender mainstreaming has led to a situation in which many political issues are discussed in terms of gender equality. Thus, violence against women has been on the Swedish political agenda for the last 30 years and is articulated as a severe gender-inequality problem [2]. Gender equality thus holds a prominent position and is the major way in which violence against women has been articulated politically. Since we want to map out the dominant discourses to which the healthcare sector has to relate to, it is important to analyse how violence is articulated in gender-equality documents. Different ways of framing or articulating the problem might lead to different understandings of how healthcare providers see their role in relation to victims [18].

### Focusing on three interrelated areas

In order to broaden the understanding of the healthcare sector’s readiness to deal with violence against women, we do not only analyse the healthcare sector per se, but use an interdisciplinary approach (i.e. feminist legal studies, public health, and political science), with the aim to situate the healthcare sector within its political and legal context. To achieve such a comprehensive view in this paper, we focus on three main questions regarding violence against women: (1) How is the healthcare sector governed legally and what is the legal obligation for the sector? (2) How are public health interventions and preventive actions framed in public health policies? and (3) How is the violence articulated and understood in healthcare law and policy as well as in gender equality policies?

### Gender and violence

The theoretical framework for the analyses lies within feminist theory, viewing violence against women as contributing to maintaining the order of unequal and gendered power relations that encourage or excuse violence [19]. Using such a feminist critique of violence against women means that it is not enough to measure force and number of slaps and acts, but to view violence as part of larger societal structures that maintain power hierarchies with male dominance and female subordination [20–22]. Women seldom initiate violence, but usually practise it as self-defence and out of fear of their partners [23]. When comparing men and women subjected to violence by a former or present partner, women are repeatedly and more seriously abused, both physically and sexually, resulting in more severe consequences for their health and wellbeing [23]. We use the terms ‘men’ and ‘women’ notwithstanding that these are constructed and unstable categories that get their meaning within a heterosexual framework.

There is substantial variation in research and policies regarding the terms used for different forms of violence against women. Our starting-point and subject for analysis is the broad term “violence against women” which includes a variation and continuum of different types of violence, contexts and relationships to the perpetrator. When relevant we use other or more specific terms, for example the terms used in the documents we analyse. We also use the terms perpetrator and victim as used in the legal system, although we know that such descriptions can be questioned; for example, victims are also survivors [24–26].

### Discursive policy analysis

Our starting point is that the official rhetoric in areas such as public health, gender equality and law is crucial for all work in the public sector related to violence. We work with discursive policy analysis, which means that we understand that the way in which a policy is articulated or framed has consequences for the prospects of solving the problem, and participates in constructing the issue [27, 28]. The policy process is not at all straightforward; suggested solutions might lead to new and unintended problems. Added to this, political problems are not merely pre-existing *outside* of the policy-making process, but rather are constructed within policy processes [29]. Policies and laws on violence against women can be influenced by different competing discourses regarding causes and possible solutions. Policies and laws that may appear rather similar can become fundamentally different, depending on how an issue is framed, named and made meaningful [30]. Discursive struggles are common when establishing an understanding of the problem or when finding the most suitable solutions and what implications they might have when it comes to the healthcare sector’s responsiveness. Correspondingly, this leads to varying consequences for victims exposed to violence. We therefore analyse how violence and public health are framed and made meaningful in these policies and legal documents. For example, it makes a difference if the policy problem is framed as a matter of “family violence” without connecting it to a gender perspective, since the magnitude of the problem as well as the perspective on who is a perpetrator and who is a victim might get lost, and consequently there is a risk of reducing the problem. This means that when we conduct the analyses, we first look for how the problem is framed, then we look for the proposed policy solutions and finally discuss their likely effects from a feminist theoretical perspective [29, 30].

## Context and methodology

This article stems from a multidisciplinary research project in which we investigate the Swedish healthcare sector’s governance and readiness for dealing with men’s violence against women framed as a public health concern. Around 84% of the healthcare in Sweden is publicly funded through the taxation system [31], and the current study concentrates on this public sector of healthcare. The healthcare sector is organized through 21 regions, which all have the primary responsibility to fund and provide healthcare to the population within their region. Much of the political decision-making about healthcare and governing of the sector is conducted at the regional level due to strong regional self-government. However, the Swedish state has become increasingly active in governing and regulating the healthcare sector through a mixture of top-down and interactive methods. The current article focuses on *national governance* through healthcare law and policies. These laws and policies provide the basis and starting points for regional governance and give a frame of reference for what the regions are obliged to do and what they must adhere to [32].

The most important governance documents representing the public health turn have been interpreted in a multi-disciplinary manner. It is our ambition that the diversity of material and approaches will broaden the scope and highlight different aspects of the public health turn on violence against women. Therefore, the legal documents have primarily been analysed from a feminist legal point of view, the public health actions and interventions have been analysed from a public health perspective and the general gender-equality policies have been analysed from a policy angle. The feminist and discursive approaches described above and analytical questions described below unite the analysis at a general level.

The methodological approach to law is socio-legal [33] with an analytical focus on the interaction between the internal processes of law and the healthcare sector. The legal material has been analysed in two steps. First, a doctrinal legal method is used to describe how the sector is governed legally and to identify the main content of the legal obligation. Doctrinal legal methods differ between countries but have in common the idea that the law can be correctly and objectively described and fixed based on specific authoritative legal sources such as legislation and case law [34]. Here, only legally binding regulations and non-binding guidelines issued by the National Board of Health and Welfare are analysed because national healthcare legislation lacks provisions addressing violence and the legal obligations of the healthcare sector cannot be subject to adjudication in court. Second, how the legal obligation is constructed is problematized using a discursive policy analysis approach.

In order to analyse how the violence is articulated and understood, we have worked with methodological questions emanating from our discursive policy analysis approach. The analytical questions are:

- How is the violence described and what is regarded to be the problem?
- What solutions and interventions are suggested in order to solve the problem?

These questions are discussed in relation to the legal regulations and general gender-equality policies as well as in relation to more specific public health documents. The documents included in that analysis as well as in the analysis of public health actions and interventions are reports from official inquiries, government bills, government action plans on gender equality, binding regulations, and guidelines, mostly from around 2010 and onwards.

The Swedish parliamentary system is built on the ideal that governmental law proposals and policies should be underpinned by a thorough investigation, an official inquiry (SOU) led by a public investigator who is given the task of carrying out the official inquiry by analysing the problem and coming up with solutions. A central document representing the public health turn is SOU 2014:49, Våld i nära relationer – en folkhälsofråga (Violence within Close Relationships – a Public Health Issue) [4]. This 2014 report was the result of a mission given to a national coordinator, appointed by the Government, to coordinate different authorities to deal with and prevent violence within close relationships.

## Analyses

### Legal governing and legal obligation

#### Governance through framework legislation and administrative regulations

The Health and Medical Services Act is to large extent framework legislation. It gives a legal frame and leaves the filling in of this frame to public bodies and administrative authorities. Framework legislation provides the parameters against which the activities can be measured [35], such as the goals of the healthcare sector, and the duties of healthcare providers and personnel. The aim is to promote material justice and stands in contrast to the traditional rule of law, with its individual rights and a formal notion of justice [35]. Consequently, healthcare legislation has no counterparts in terms of individual legal claim rights [36] that an abused woman can bring to court to enforce her rights or be compensated because her rights have been violated in her encounters with healthcare.

The Health and Medical Services Act contains no provisions regarding violence. The need for a provision focusing on the needs of abused women, has never been a subject for discussion in preparatory works for healthcare legislation. Proposals regarding children [4] and violent men [37] have been presented but has either not been acknowledged in later legislative processes [38] or found unsuitable for regulation [37]. The National Board of Health and Welfare (the Board) has a mandate to draw up legally binding regulations that are designed to enhance quality and safety in health and social care. The Board also issues guidelines on how legislation and regulations can or should be implemented. The lack of national legislation and the character of framework law makes these regulations and guidelines the most important normative sources through which the sector is nationally governed, and the legal obligation is constituted.

#### The legal obligation

The first guidelines on men’s intimate partner violence against women were issued by the Board in 2009. However, these guidelines did not address the healthcare sector, only the social services. In 2014, binding regulations and new guidelines that are still in force replaced the 2009 guidelines. The 2014 Regulations and Guidelines [5] address both the social services and the healthcare sector. As a complement to the 2014 Regulations and Guidelines, the Board has also published a handbook [39]. The aim of this 2016 Handbook is to support municipal social welfare boards and healthcare providers regarding how to organize their work at an overall level and to deal with individual cases of violence. In 2014, the Board also issued guidelines on when and how the social services and healthcare sector should routinely pose questions about exposure to violence in order to enhance the preconditions for discovering violence [40]. These guidelines have later been developed and integrated into the 2016 Handbook.

Unlike their predecessors, the 2014 Regulations and Guidelines and the 2016 Handbook are gender neutral. A new concept, “violence within close relationships” (*våld i nära relationer*), is used as the main concept for the violence covered by the documents; that is, children witnessing violence in intimate relations, victims of intimate partner violence and victims of violence from other relatives, regardless of gender or age. Consequently, the documents have a wider target group than before. Another novelty is that the guidelines also cover support offered to perpetrators of violence.

In the absence of a specific obligation regarding violence in national healthcare legislation the Board refer to two other legal foundations for the regulations [39]. First, international legal obligations for Sweden according to conventions such as the CEDAW and Istanbul conventions, and second, two provisions in the Health and Medical Services Act [32] that are generally formulated: Chapter 3 section 1, which stipulates good health and good care on equal terms and conditions for the population as the goal for the healthcare sector, and chapter 5 section 1, stipulating that healthcare shall be conducted in a way that fulfils the demands for good care. The obligation is thus constituted through an extensive interpretation of the Health and Medical Services Act and constructed as an obligation to provide good healthcare for victims of violence within close relationships.

How then is the obligation to provide good healthcare for victims of violence within close relationships constructed in more detail? The 2016 Handbook states that the task is to *examine and treat* the victims and to do so to the extent that the specific competence of healthcare personnel is needed. The 2014 Regulations [5] contain one provision that specifies the obligation to provide healthcare for adult victims (Chapter 8 section 9). First, in cases where symptoms or other signs incite a suspicion that the patient is or has been exposed to violence or abuse by a close family member, the healthcare provider is obliged to make sure that healthcare personnel ask the patient in private about the cause behind the symptoms or signs. Second, if the suspicion remains after these questions, the healthcare provider is obliged to make sure that healthcare personnel: (a) inform the patient about the opportunities to get healthcare or support from social services or non-governmental organizations, and (b) consider what physical and/or psychological needs connected to the violence the adult person might have. Besides this obligation to provide good healthcare, the 2014 Regulations mainly contain obligations regarding management systems, routines, documentation and cooperation. The 2016 Handbook [39] provide no additional information about what is included in the obligation or how it is supposed to be carried out.

The 2014 Regulations also contain two guidelines that are not legally binding. One recommends that the healthcare provider ensures that healthcare personnel have enough knowledge about violence and abuse within close family relationships. The other concerns the issue of routinely posing questions about exposure to violence to enhance the preconditions for discovering violence. The 2016 Handbook describes it as a task or role for the healthcare provider to discover and notice violence. This issue will be dealt with in the next section.

#### A self-evident obligation regarding an evasive problem

The articulation of violence against women as a public health issue is weak in legal documents. The documents only touch upon the third public health intervention, presented later in this article, namely treatment and rehabilitation when ill-health has already occurred. Therefore, the violence is mainly represented as an ‘ordinary’ healthcare issue centred on curing the individual patient, without considering issues of health-promotion or prevention, individually or on group level.

The main obligation and the way in which it is specified raise questions. Is it not superfluous to state that victims of intimate partner violence who turn to the healthcare sector shall be provided with good healthcare in relation to their physical and psychological needs? Is it not self-evident that healthcare personnel must have enough knowledge to do their job; for example, to ask about the causes behind symptoms or injuries? When pushed to the very limit, the only additional obligation that has been introduced in these legal documents is the obligation to refer victims with needs other than medical, to the relevant agencies. It can be questioned if and to what extent Swedish healthcare law, as expected by WHO [24], expand the sector’s role in regard to violence protection.

When we approach the legal documents discursively, we can see that they express a need to rectify a problem without explicitly formulating what that problem is. The way in which the problem is said to be rectified suggests that it consists of shortcomings in providing good healthcare for victims, but without formulating what these shortcomings are, apart from a lack of asking about violence. Neither is the meaning of ‘good healthcare’ for victims specified beyond the issue of asking about violence, nor are any special needs for such victims in general, or women exposed to male partner violence, acknowledged.

On a general level the problem is constructed as shortcomings for the sector in realizing that violence within close relationships can be a cause behind why people turn to healthcare, and that the sector is an important public actor in discovering such violence. Thus, it is expressed more as a general obligation towards ‘society’ than as an obligation towards the victims of violence. Such a discourse contributes to why it is difficult to define what the obligations are in relation to the victims of violence.

In all, the legal documents construct the obligation to deal with the violence as ‘ordinary’ healthcare and express no ambition to provide anything ‘special’ or ‘new’ to meet the particular needs of women exposed to male partner violence, besides a rather cautious message about sometimes asking about violence.

### Framing of violence in public health policy, interventions and preventive actions

Public health interventions are supposed to target individuals as well as groups of people, but societal institutions are also obliged to promote health and prevent ill-health at the societal level. Sector coordination is emphasized. In order to improve the health of a population, three different means are available: a) health-promotion interventions, which refers to improving or protecting people’s health while there is still no sign of ill-health; e.g. strengthening people’s physical, mental and social well-being and supporting healthy behaviours; b) prevention of ill-health, meaning reducing risk factors for ill-health, e.g. supporting patients in healthcare to make good, healthy choices, screening for diseases and the prevention of injuries; c) treatment and rehabilitation when ill-health has occurred, in order to minimize further health problems, e.g. medication, treatment programmes etc. The Swedish Association of Local Authorities and Regions emphasizes that healthcare institutions play an important role in public health interventions, but that they should also include and engage wider sectors of society. A so-called ‘health-promoting healthcare’ must focus on an organization’s total interventions that contribute to improved health for individuals as well as the population as a whole, and must include a wide range of interventions, not just the treatment of diseases [32].

In the analysis of the public health documents, we found three central framings, of which one represents the problem formulation, and two represent suggested solutions to the problem.

#### Violence against women is a severe societal and public health problem

The overall formulation of the problem is simply that violence against women is a severe public health problem, as well as a societal problem. In the 2014 report from the national coordinator and the 2016 Handbook, violence against women is described and articulated as a problem that causes a variety of health problems, such as physical and psychological [4, 39]. Besides human suffering, it is also stated that violence against women causes considerable societal costs. Further, in the 2014 report and 2016 Handbook [4, 39], the violence is linked to gender equality, as it is regarded to obstruct exposed women from accessing their human rights and freedom. This, it states, will thus place high demands on society’s preventive work among a variety of actors, but it also requires action to detect early signs of exposure to violence among both children and adults.

#### Daring to ask about violence in therapeutic encounters

Even though we have seen a public health turn on violence against women in Sweden, the interventions have not been straightforward and, in general, few women subjected to violence have been identified within Swedish healthcare. Daring to ask is the most prominent suggested solution, and it comes back in all the reports. When the Swedish Board of Health and Welfare presented a first outline, it was suggested to only ask about violence when there is already a suspicion. The Board argued about this limited recommendation as an ethical issue and also claimed that it was no strong scientific evidence about inquiring as resulting in less violence and/or improved health. However, this led to a debate and opposition by Swedish researchers in Swedish media [41] which made the final 2014 guidelines to be somewhat revised and expanded [40]. Besides the general obligation for health professionals to ask when there is a suspicion [5], the 2014 guidelines also recommended to routinely ask about violence, although still highly selective, and cover only patients in three distinct healthcare sectors: (1) women in antenatal care with recurring contacts with a midwife; (2) women in psychiatric care; (3) children and youth in psychiatric care [40].

The 2014 guidelines include directions about asking patients about violence, the importance of certain responsibilities, and collaboration between different actors within, and outside of healthcare; all in all, with the aim to increase the patients’ opportunities to disclose the existence of violence. Furthermore, it emphasizes the need of care and support for women and their children exposed to violence [40]. When the guidelines were integrated into the 2016 Handbook [39], the target group for asking was widened from women and their children witnessing violence to including both victims and perpetrators regardless of gender and age. Moreover, more detailed guiding principles were introduced, such as the prerequisites to ask and how to state questions about violence.

#### A national goal for public health addressing violence against women is needed

A national goal for public health is suggested in the 2014 report in order to highlight the seriousness of the problem with violence as a public health issue. The reason is the urgent need to highlight violence against women, as it causes so much ill-health. As public health deals with creating societal prerequisites for good health on equal terms for the entire population, it is essential that violence within close relationships is included in the national goals for public health. The report also alludes to the gender-equality policy, in which it is stated that men’s violence against women must come to an end. The suggested new goal is supposed to increase efficiency in the healthcare system, sharpen interventions, increase quality in care and add a more holistic perspective on patients [4].

In sum, the problem is rather loosely defined, and suggested interventions are scarce and somewhat vague. For instance, the Board emphasizes preventive work of different kinds, such as healthcare’s ability to achieve the early detection of those women and men at risk, and preventive work with perpetrators, but it is not entirely clear to what extent the Board distinguishes between different forms of public health interventions. Preventive work in healthcare for the detection of exposure to violence is specifically prioritized, but not explained how they are going to be executed. Therefore, we judge that the solutions of public health interventions are rather vaguely expressed and defined. The recommendations lack substantive details on how a public health perspective should be integrated into healthcare work, with one exception: the issue of daring to ask about violence. The prevention of violence in the form of routine enquiry about violence for specific groups of patients seems to be the main recommendation. We draw the conclusion that this is mainly secondary prevention, i.e. the early detection of risk factors and actual violence, as well as working with perpetrators to not repeat a violent behaviour. In order to strengthen the readiness to routinely ask about violence, the key is to be prepared by regular training, developed guidelines, networks for support and links to referrals [42, 43]. In addition, we judge the suggested new national goal for public health; namely: “freedom from violence within close relationships”, to be important as there has been criticism of the national goals for public health for not including violence as a public health matter. The suggested public health interventions are however mixed with all sorts of arguments that blur the main message of gender-equality policy of ending men’s violence against women.

### Varying articulations and comprehension of violence

In this section, we highlight how violence against women has been included and articulated in Swedish gender-equality policies, public health policies, and legal documents (already discussed above). In our analysis of the documents, we have found three major ways of conceptualizing the problem – the first is to describe it as a problem of men’s behaviour (men’s violence against women), which is most common in gender-equality policy; the second is a fragmentation of the violence into different types; and, finally, the articulation “violence within close relationships” which seems to be the dominant way of understanding the problem in public health policy and law.

#### ‘Men’s violence’

When scrutinizing national public policy on gender equality, one could say that in general after 2000, the Social Democratic-led governments have underlined the ‘power imbalance’ between women and men and stated that their understanding is ‘feminist’ [12], whereas the liberal-conservatives have not necessarily labelled their reforms as feminist. Both political sides have, however, focused a lot of efforts against violence as part of their gender-equality politics. Violence against women has been by far the most prioritized gender-equality issue in Swedish politics for decades, both in terms of commitment and resources:

Violence and other forms of abuse against women is today, according to the government, the most urgent issue when it comes to gender equality. Psychological, physical, and sexual violence can under no circumstances be accepted or condoned. To counteract men’s violence against women is therefore of highest priority for gender equality politics (Our translation, p 23) [44].

Thus, there are slightly different discourses as to how to understand this violence, and in the following we present these ways of articulating and explaining violence.

One of the more prominent ways to conceptualize violence in gender-equality policy is to describe it as “men’s violence against women” and as a matter of gender and power. The introduction of this perspective, that is to point out that men are perpetrators, has been described as a ‘radicalization’ of the Swedish political agenda [45]. In 2005, in line with the framing of violence as ‘*male* violence’, the national gender-equality goals were reformulated by the government as: “Men’s violence against women shall come to an end” [46, 47]. The reason for replacing the former goal was that it was considered to be framed in gender-neutral terms (‘gender-related’ violence), and the government wanted to point out that the perpetrator is a *man* in most cases [47].

This articulation of violence against women as being gendered, but as a problem of *men’s* behaviour and masculinity, has been on the agenda since then. In the government bill of 2005, it was stated that “society has to take responsibility for all victims of violence, no matter who is the perpetrator and the victim. The task of *gender-equality politics* is, however, to counteract men’s violence against women” [47], see also [48]. This led to a situation in which the government was criticized for having a hetero-normative perspective and marginalizing lgbtq persons and not considering violence in same-sex relationships. A feminist perspective has also recently been stressed:

Men’s violence against women is a serious and extensive societal problem that causes immense physical and psychological suffering. It is the ultimate consequence of the prevailing power imbalance between women and men. Perceptions of gender, power and sexuality have an essential significance for all forms of men’s violence against women [ … ] The government’s goal that men’s violence against women shall come to an end also includes honour-related violence and oppression as well as prostitution and human trafficking for sexual purposes (Our translation, p. 33) [12].

Even though the term ‘men’s violence against women’ has been introduced and is defined as being a more extensive conceptualization, in which honour-related violence is included, there has been a tendency to separate violence into different types, as will be further discussed below.

#### The fragmentation of violence

In 2007, the liberal-conservative government presented the *Action plan to combat men’s violence against women, honour-related violence and violence in same-sex relationships* [49]. Here, the government separates honour-related violence from men’s violence against women, and also includes violence in same-sex relationships, which had been neglected up until then. This meant that the specificities of different situations and structures could be discussed. However, this shift has been described as ‘fragmented violence’ and has been criticized [50]. One of the problems is the separation of honour-related violence from men’s violence against women. When the concept of honour-related violence was introduced, it was primarily conceptualized by referring to *culture* as an explanation. Honour-related violence was framed as ‘patriarchal’ and ‘new’ to Swedish society. It was seen as having a cultural motivation, and as something that emanated from outside Sweden, from a particular cultural and geographical region, primarily the Middle East [51]. The earlier feminist perspective on violence was seen as obsolete, and as having almost no explanatory value, since *culture* was considered to be the major way to explain this particular form of violence. Violence occurring in ‘Swedish’ families could now be disassociated from patriarchal structures [52]. The focus on men, and the argument that violence against women is a problem of masculinity, was thus downplayed. As a consequence, there are separate ideas about how to understand so-called honour-related violence as compared to ‘men’s violence against women’ (which is then indirectly understood as being perpetrated by ‘ethnic Swedes’, but not having to do with Swedish culture) and violence in same-sex relationships.

#### Violence within close relationships

Yet another way of articulating the problem is to label it as ‘violence within close relationships. This is the framing used in public health policies and legal documents and is more common in the departments of justice and social affairs, while it is only occasionally used in general gender-equality policies. This framing can be traced back to 2012 when the liberal-conservative government appointed the national coordinator against violence within close relationships, with the task of coordinating different authorities to prevent violence [4]. It is noteworthy that, in this setting, there is a tendency for the problem to be made gender neutral. The 2014 Report talks about ‘victims of violence’, without gendering either victims or perpetrators. This also means that the goal of gender equality is not necessarily stressed, and that the structural aspects of the problem are downplayed.

The 2014 Regulations and Guidelines [5], and the 2016 Handbook [39] also articulate the violence as gender neutral. This shift in legal documents, from a gendered frame in previous guidelines to a gender-neutral one in the binding regulations, is what normally happens when a social problem is transformed into binding law through being made a juridical matter. The liberal legal discourse on formal gender neutrality as the only acceptable method for constructing law outweighs reasons for gender awareness, sensitivity or specificity in the law, however strong these reasons might be from other perspectives [53]. However, the 2016 Handbook also mentions that, while both men and women are exposed to violence within close relationships, women are more exposed to recurring and more severe forms of violence than men. This is a public health problem that can cause severe consequences for those exposed; it is concluded [39].

In 2018, the report of an official inquiry presented solutions on how to break men’s violent behaviour within close relationships, and in this setting the problem was named ‘violence against related persons’ (Våld mot närstående), thus including honour-related violence and violence within same-sex relationships [37]. One of the suggestions was to create a national centre working with perpetrators, and the investigator also pointed especially towards the healthcare sector as being responsible.

The articulation of violence as a problem occurring within close relationships makes it (at least in theory) possible to include men’s violence against women, honour-related violence and violence within same-sex relationships. However, this framing has more recently been criticized for being too narrow in another sense:

Only focusing on violence within close relationships means that many other aspects of violence against women, for example rape and sexual abuse of girls and women by an unknown perpetrator, work-related violence, sexual harassment, grooming, prostitution and human trafficking for sexual purposes, is made invisible (Our translation, p. 52) [54].

Thus, to sum up, when the problem is articulated as a matter of violence within close relationships, the link between violence and gender-equality politics might be downplayed. That is, there is a risk that, on a more societal discursive level, the gender-equality perspective is not considered a part of violence against women as a public health problem. Another risk is that so-called honour-related violence might be excluded from this framing.

## Methodological considerations

The multi-disciplinary approach in this paper opens up space for scrutinizing the ‘public health turn on violence against women’ from various angles. The team consists of researchers from feminist legal studies, political science and public health sciences, all with a focus on gender as a central component of analyses, and with violence against women as their specific field of research. By taking this approach, our intention has been to provide a more comprehensive understanding of the phenomenon than would be possible if it was scrutinized in separate analyses. The current research team has worked together for more than 10 years and we have gradually learnt how to integrate knowledge and perspectives from the different disciplines in order to analyse law and policy in relation to the public health turn on violence against women. Although the respective researchers from each field have taken major responsibility for their section, we have made a joint effort to integrate and discuss the analysis as far as possible. Accordingly, the analyses and the conclusions presented here, is a negotiated outcome that can be viewed as inter-disciplinary collaboration.

## Conclusions

Violence against women is increasingly seen as a public health problem in Sweden. We were interested in analysing *how* policies were framed in this turn towards public health. One of our main findings is that in law and public health policies the problem is primarily articulated as a matter of “violence within close relationships”. The term “violence within close relationships” is a new approach that deviates from the earlier framings of “men’s violence against women”, and is a specific Swedish policy term. This new approach indicates a gender-neutral conceptualisation in which both victim and perpetrator are invisible in terms of gender. Another main finding is that the legal obligations and the problems for the healthcare sector are only vaguely defined. Some of the vagueness may have to do with a lack of legal governance. The rather far-fetched interpretation of the concept of ‘good healthcare’ as being what establishes the healthcare sector’s legal obligation provides only weak guidance for how to understand and formulate the obligation in more detail, which in turn impacts upon the healthcare providers’ and healthcare personnel’s ability to develop their work. Our interpretation is that this lack is reflected in the cautious and rather poorly implemented ‘daring to ask’ guideline.

The healthcare sector’s political and medical autonomy usually leads to the conclusion that it must be left to the sector itself to decide how to deal with health problems. However, it is rather usual that national guidelines exist, for example regarding diabetes, heart diseases and psoriasis, and binding regulations can be found, for example regarding blood transfusion. Our interpretation is that the mere fact that regulations and guidelines have been issued gives evidence of a perceived shortcoming regarding how the sector previously conceived of, and dealt with, violence within close relationships. In our view, this calls for a discussion about how the legal governance could be developed. Should a clearly formulated and informative legal obligation be introduced into the Health and Medical Services Act? Is there a need for more far-reaching and detailed binding regulations about how healthcare personnel should perceive violence against women and act when encountering it?

The vague conceptualization of what is meant by the stated fact that violence against women is a major public health problem may result in healthcare institutions struggling to implement guidelines and action plans. Which groups should be screened for violence? On what grounds are healthcare professionals supposed to ask about violence? How often should patients be asked about exposure to violence? And is asking about violence the only means for healthcare providers to address this major public health problem? It is proposed in the policies that, in order to reduce violence, there is a need for both small-scale and large-scale interventions, and that this must be long-term sustainable work including the entire society and improved coordination between criminal, gender-equality, social, and public health policies.

In general gender-equality policies, violence against women is articulated as one of the most severe problems and a powerful example of the lack of gender equality. Yet, gender equality is relatively invisible in both the public health and legal documents regulating the healthcare sector. The same goes for human rights. This means that the healthcare sector might be unaware of its role in Swedish gender-equality policy and the sector’s obligation to ensure women’s human rights. The question is, of course: how, and whether, do professionals working in the healthcare sector perceive initiatives against violence within close relationships as being a matter of gender equality and women’s human rights?

The lack of a gender-equality discourse is related to and amplified by the de-gendering of violence against women in the steering documents for the sector. Hence, the problem that the healthcare sector is supposed to deal with risks becoming reduced to healthcare personnel’s individual encounters with victims whose gender is of no significant importance. However, gender neutrality is not enough to fulfil international legal human rights obligations regarding men’s violence against women. As pointed out by the UN special rapporteur on violence against women, a shift towards gender neutrality hides the fact that violence against women is a system of domination and a systemic, widespread and pervasive human rights violation, experienced largely by women because they are women [55]. Neither does gender neutrality fulfil the obligations of the Istanbul convention, as shown in several Council of Europe evaluations [56, 57] and problematized as bearing the risk that gender insensitive interventions lead to gaps in protection and support and contribute to the re-victimisation of women [58].

In summary, our analysis shows that, while violence against women in some policy documents is clearly framed as a public health problem, such a framing is absent in others, or is transformed into a gender-neutral problem of violence within close relationships. Moreover, it is not yet clearly articulated in law or policies what the framing should lead to in terms of the healthcare sector’s obligations, interventions and health promotions, apart from an equivocal discourse on daring to ask about violence. In the latest gender-equality policy document, the importance of the healthcare sector in striving towards the gender-equality goal of eliminating men’s violence against women is emphasized in terms of the early detection of violence, prevention and providing healthcare. This implies increasing demands on the sector, but our analysis shows that it also implies increasing demands on how the sector is governed.

In order to develop the Swedish healthcare sector's work with violence against women and in order to be able to provide recommendations, further studies are needed. For instance, studies focusing on the 21 regions of the country are important, as we believe that much of the detailed prevention programmes and action plans can be expected to be developed at that level.

## Data Availability

The article only contains analysis of official documents. They are all included in the reference list. For on-line documents, hyperlinks with the noted point in time when they were assessed, are included in the reference list.

## Abbreviations

CEDAW: Convention on the Elimination of All Forms of Discrimination against Women
NCK: Nationellt Centrum för kvinnofrid [The Swedish National Centre for Knowledge on Men’s Violence against Women]
SOU: Statens Offentliga Utredningar [The Government’s Official Inquiries]
UN: United Nations
WHO: World Health Organization
